# Macromycetes Under Pressure: Diversity and Species Composition Across an Urbanization Gradient in the Puebla-Tlaxcala Valley of Mexico

**DOI:** 10.3390/jof12060397

**Published:** 2026-05-30

**Authors:** Marko Gómez-Hernández, Etelvina Gándara, Eder Dorantes-Marín, María Toledo-Garibaldi

**Affiliations:** 1 SECIHTI-Facultad de Ciencias Biológicas, Benemérita Universidad Autónoma de Puebla, Av. San Claudio S/N Col. Ciudad Universitaria, Puebla 72592, Puebla, Mexico; 2 Facultad de Ciencias Biológicas, Benemérita Universidad Autónoma de Puebla, Av. San Claudio S/N Col. Ciudad Universitaria, Puebla 72592, Puebla, Mexico; ledm.bio25@gmail.com; 3 Red de Ecología Funcional, Instituto de Ecología, A.C. Carretera Antigua a Coatepec No. 351, El Haya, Xalapa 91073, Veracruz, Mexico; maria.toledo@inecol.mx

**Keywords:** macromycetes, urbanization, species diversity, species distribution, macromycete communities, oak forests

## Abstract

Macromycetes are of great relevance to the functioning of terrestrial ecosystems, but habitat transformation can significantly alter the structure of macrofungal communities. Urbanization is regarded as a major threat to biological diversity; however, knowledge of its impact on macromycetes remains scarce. The present study aimed to assess diversity and distribution patterns of macrofungal species across an urbanization gradient in the Puebla–Tlaxcala Valley of Mexico and determine the effect of urbanization on macromycete communities. From May to October 2024, macromycetes were collected in four oak forests. Fungal specimens were classified based on their macromorphological and micromorphological characteristics. Topographic (1), microclimatic (4), vegetation structure (5), environmental (2), and urbanization (4) variables were included in the analyses. A total of 296 macrofungal species were recorded. Diversity has been shown to decline with increasing urbanization. Species composition shifted across the gradient, with the most urbanized sites showing higher turnover. The interplay of microclimate, vegetation structure, and urbanization was a key driver of the observed patterns, underscoring the sensitivity of macromycetes to urban environmental change. The findings highlight the importance of microclimatic buffering and habitat continuity for sustaining macrofungal communities within urban areas.

## 1. Introduction

Urban growth poses significant challenges for biodiversity conservation, as it has dramatically transformed landscapes and is regarded as a major threat to biological diversity [1,2,3]. Urban areas are recognized as ecosystems shaped by anthropogenic disturbance, including novel species assemblages, altered disturbance patterns, and significant environmental gradients such as urban heat islands and pollution levels [4,5]. Currently, approximately 4.2 billion people live in urban environments worldwide, and projections suggest that this trend will persist, reaching 6 billion urban inhabitants by 2045, representing nearly 70% of the world’s population [6,7,8]. In Latin America, 82% of the population lives in urban areas, including megacities (over 10 million residents). Mexico reflects this trend, with approximately 88% of its ca. 132 million people residing in urban areas [9]. Global research on urbanization indicates a decline in species numbers, with human impact on biodiversity decreasing from city centers to wild areas. This may result in differences between urban and surrounding non-urban areas regarding the structure of community groups such as birds, plants, insects, and fungi [10,11].

Macromycetes (fungi that produce sporomes visible to the naked eye) are a highly relevant group due to their roles in the functioning of terrestrial ecosystems as organic matter decomposers, mutualists, and pathogens [12,13,14]. Decomposer fungi contribute to the nutrient cycle and soil formation; mycorrhizal fungi establish symbiosis associations with plants and contribute to the recruitment of seedlings and the growth of trees; and pathogenic fungi play an important role in regulating populations of plants, animals, and fungi [15,16,17]. The diversity of fungal species is estimated at ca. 6.2 million globally [18], and between 53,000 and 111,000 represent macromycetes [19]. For Mexico, estimates suggest more than 250,000 species of fungi, and approximately 9000 to 11,000 species of macromycetes [20].

Given the relevance of these organisms to ecosystem functioning, understanding how microclimatic, environmental, and vegetation factors relate to the diversity and distribution of macromycete species is a growing interest in ecological research [21]. Studies on the diversity and composition patterns of macromycetes in temperate and tropical forests suggest that species richness and sporome production are mainly related to topographic and microclimatic factors [21,22,23,24]. Also, vegetation structure and tree species composition can affect macrofungal communities by influencing the amount and quality of resources provided by plants [22,25].

Fungal communities are extremely vulnerable to habitat loss caused by urbanization and other anthropogenic activities. This often leads to a decline in species richness and alters species composition in various habitat conditions, mainly influenced by microclimate, and factors related to substrate availability and vegetation structure [10,11,26,27]. The impervious surfaces in urban areas can reduce soil’s water absorption and impact microclimate [26], and the urban surface capacity to absorb solar radiation and the heterogeneous distribution of vegetation can significantly increase temperature owing to the heat island effect [28,29], causing a decrease in macromycete richness and sporome production [30,31]. However, most ecological studies about macromycetes focus on conserved forests, and little is known about the ecology of macrofungal communities in urban areas [26]. In Mexico, there is only one study that analyzes the diversity and distribution of species along an urban gradient. This study found that functional diversity and species richness decline with increasing urbanization, and there is high turnover in species composition across the study area, which is linked to microclimatic and urbanization factors. Additionally, their results indicated that some of the available resources in the niche space within the most urbanized sites are not being utilized [27].

The Puebla-Tlaxcala Metropolitan Zone (PTMZ), located in the Puebla-Tlaxcala Valley, is the 4th most populous metropolitan zone in Mexico, with ca. 3.2 million inhabitants. About 87% of the population resides in the State of Puebla, and 13% resides in the State of Tlaxcala, with 95% of the total population in the PTMZ located in urban areas [32]. However, no studies have been conducted to evaluate the variation in species diversity and composition among macrofungal communities in relation to environmental or urban variables in the States of Puebla and Tlaxcala. Most mycological studies in Puebla address the use of wild mushrooms within rural communities, the commercialization of sporomes in local markets [33,34,35], and some studies focus on developing species inventories [36,37]. Similarly, in Tlaxcala, most studies focus on ethnomycological topics, with some exploring the taxonomic diversity [38,39].

In this context, the present study aimed to evaluate variation in the diversity and composition of species in macrofungal communities along an urban ecosystem in the Puebla-Tlaxcala Valley of Mexico, and identify how microclimatic, environmental, urbanization, and vegetation structure factors relate to the observed variation. The hypotheses related to the aim of the study were: (1) macrofungal diversity declines as the urbanization increases, showing higher diversity in less urbanized areas, (2) microclimate and vegetation structure are the main drivers of diversity variation along the study area, and are also related to the variation in species composition, and (3) the turnover of species composition is lower between the least urbanized sites and higher between the most urbanized areas.

## 2. Materials and Methods

### 2.1. Study Area

**Figure 1:**
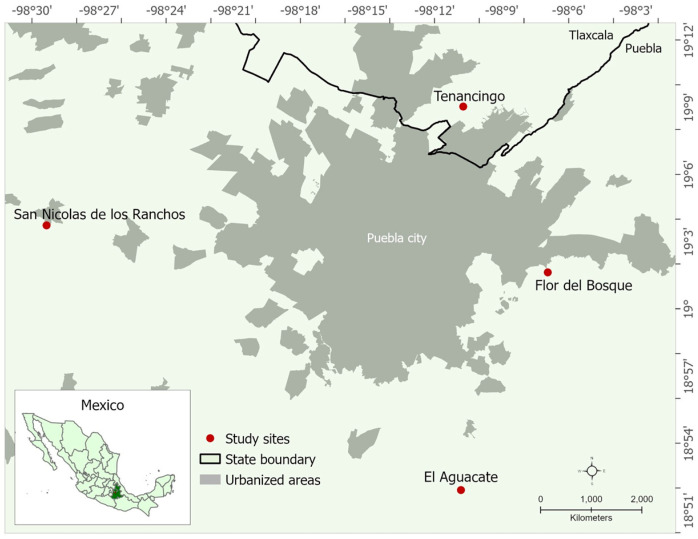
Location of the study sites in the Puebla-Tlaxcala Valley of Mexico. Image by M. Toledo-Garibaldi.

The study was carried out in the Puebla-Tlaxcala Valley of Mexico. Four areas with predominant oak vegetation and varying urbanization levels were selected in localities surrounding the city of Puebla. Flor del Bosque State Park (Site 1) at 2264 m asl, protected area located within the city of Puebla, surrounded by streets and buildings; El Aguacate municipality (Site 2) at 1922 m asl, surrounded by deforested areas and unpaved rural tracks; Tenancingo municipality (Site 3) at 2237 m asl, surrounded by crop areas and unpaved rural tracks; and San Nicolás de los Ranchos municipality (Site 4) at 2616 m asl, surrounded by crop areas, unpaved rural tracks and buildings. The selected sites were as similar as possible in vegetation type, terrain topography, and understory coverage to minimize significant differences that could mask the effects of the variables used to explain the diversity and composition variation. At each site, 10 permanent plots of 10 × 10 m were established, with a distance of approximately 10 m between plots [40] (Figure 1). Approval to conduct the fieldwork in Tenancingo, San Nicolás de los Ranchos, Flor del Bosque, and El Aguacate was given verbally by the local authorities.

### 2.2. Macromycete Sampling

Sampling was carried out twice a month in every plot at each study site from May to October 2024, which corresponds to the rainy season in the region. Since macromycetes were collected in a single season, the same systematized sampling procedure was applied at the four sites to obtain comparable data useful for analyzing how macrofungal diversity and composition vary along the urbanization gradient. Each site was sampled by the same two people for 6 months, involving the same sampling effort (i.e., number of plots per site and sampling dates) in each place. Abundance was estimated as the number of collection units for each species; sporomes of the same species within a 50 cm radius, caespitose growth, fairy rings, and growing on the same trunk or branch were recorded as a single collection unit (individual). All specimens were carefully differentiated and classified at the species level based on their macromorphological and micromorphological characteristics, aided by identification keys and reference literature [41,42,43,44,45,46,47,48,49,50]. It is not necessary to know the scientific names of each species to achieve reliable results regarding diversity and distribution from the analyses performed in this study; what matters most is that specimens are accurately differentiated at the species level [12,21,27,51], thus unidentified taxa were classified into morphospecies using a higher taxonomic level approach. Hereafter, for practical purposes, morphospecies will be referred to as species. Voucher specimens are deposited in the mycological collection of the Laboratory of Fungal and Plant Diversity and Evolution, BUAP. Field sampling activities were carried out under a scientific collection permit SMADSOT.SGTDU.DGRNB 0118/2024 granted by the Secretaría de Medio Ambiente, Desarrollo Sustentable y Ordenamiento Territorial.

### 2.3. Explanatory Variables

Variables were measured within each plot at each study site. Microclimatic variables: air humidity (%), air temperature (°C), soil humidity (%), and soil temperature (°C) were recorded on each sampling date at the center of each plot. Topographic variable: slope (°) was measured once in each plot. Environmental variables: canopy openness (%) and litter depth (cm) were measured once in each plot, and at the beginning, middle, and end of the sampling season, respectively. Woody plants were counted, and their diameter and height were measured. The vegetation structure was characterized as density (individuals ha^−1^), basal area (m^2^ ha^−1^), and mean, minimum, and maximum tree height (m).

In the present study, an urbanized area refers to any location that has been altered by human activities, such as trails (roads), crops, or buildings. To describe the urbanization gradient across the study sites, explanatory variables were measured within a circular buffer zone with a 5 km radius from the center of each site. The 5 km radius was selected to allow for a considerable distance between buffer boundaries. The urbanization variables included in this study were the extent of built-up areas (km^2^), extent of crop areas (km^2^), extent of areas with introduced vegetation (km^2^), and length of streets and roads (km) [52]. All variables were derived from land-use data layers and census data from the National Institute of Statistics and Geography, and total urbanization values were calculated by buffer area using zonal statistics in ArcGIS 10.4 [53].

### 2.4. Data Analysis

The diversity of macromycetes was measured with the species richness (calculated for each site as the number of recorded species) and the first-order (1D) True Diversity index [54,55], calculated with the “entropart” package in R. Spearman’s rho correlation coefficient was used to determine the relationship between macromycete species richness and microclimatic, environmental and vegetation structure variables, using the “psych” package in R. Linear regression analyses were performed to determine the relationship between the number of macromycete species and urbanization variables along the study area, as well as the relationship between microclimatic, environmental, and vegetation structure variables and urbanization variables.

The turnover in species composition between study sites was assessed using the Chao-Jaccard similarity index [56] with the “fossil” package in R. A Non-metric Multidimensional Scaling (NMDS) analysis was performed to represent on a geometric plane the distance between study sites in relation to the composition of species, using the “vegan” package in R. The relationship between species distribution and a set of microclimatic, environmental, vegetation structure, and urbanization variables was determined by performing a Canonical Correspondence Analysis (CCA) with the “vegan” package in R. All the analyses mentioned were performed in R v. 3.2.3. [57].

A conceptual model was created to clearly visualize how urbanization, microclimate, and vegetation structure interact to influence macrofungal diversity and species composition in the study area. The model was performed in Python 3.11.5 using the NetworkX 3.2.1 library for network structure and Matplotlib 3.8.0 for visualization [58].

## 3. Results

**Figure 2:**
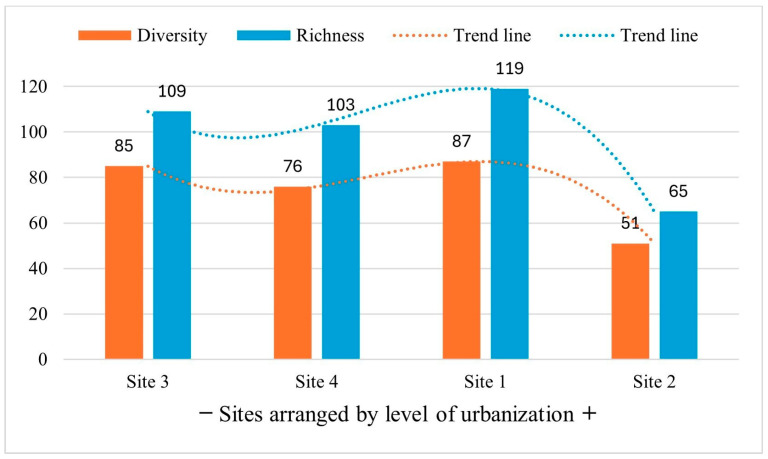
True Diversity index and species richness values in the study sites. Sites are arranged from the least urbanized (Site 3) to the most urbanized (Site 2). Dotted lines indicate the trend of diversity and species richness along the urbanization gradient.

A total of 806 macromycete individuals were recorded, belonging to 296 species (Table S1), 60 genera, and 42 families. Of the species recorded, 22 belong to the phylum Ascomycota, and 274 belong to the phylum Basidiomycota. The least urbanized site was Tenancingo (Site 3), followed by San Nicolás de los Ranchos (Site 4), Flor del Bosque (Site 1), and El Aguacate (Site 2). Flor del Bosque recorded the highest species richness (119 species), followed by Tenancingo (109 species), San Nicolás de los Ranchos (103 species), and El Aguacate (65 species). Correspondingly, the True Diversity index showed that Flor del Bosque had the highest diversity (87), followed by Tenancingo (85), San Nicolás de los Ranchos (76), and El Aguacate (51). Both the diversity index and species richness showed a similar trend of decreasing with increasing urbanization, with a peak at Site 1 (Figure 2).

Spearman’s rho correlations indicated a significant negative correlation between macrofungal species richness in the studied area and both air and soil temperature (rho = −0.5277, *p* = 0.0004; rho = −0.3419, *p* = 0.0307, respectively). Soil humidity, along with the mean and maximum height of trees, showed a positive correlation with species richness. (rho = 0.5180, *p* = 0.0006; rho = 0.4141, *p* = 0.0078; rho = 0.3152, *p* = 0.0475, respectively) (Table 1).

**Figure 3:**
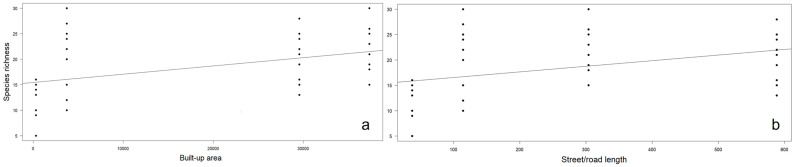
Linear regression analysis between species richness and (**a**) extent of built-up area and (**b**) street/road length along the study area in the Puebla-Tlaxcala Valley of Mexico. The fitted lines indicate a positive trend.

The linear regression analyses indicated a positive relationship between species richness and the extent of built-up areas and length of streets/roads along the studied area (r^2^ = 0.18, F = 8.28, *p* = 0.0062; r^2^ = 0.15, F = 6.5, *p* = 0.0141, respectively; Figure 3a,b). The extent of crop areas and the extent of areas with introduced vegetation were not significantly related to species richness (*p* > 0.05).

**Figure 4:**
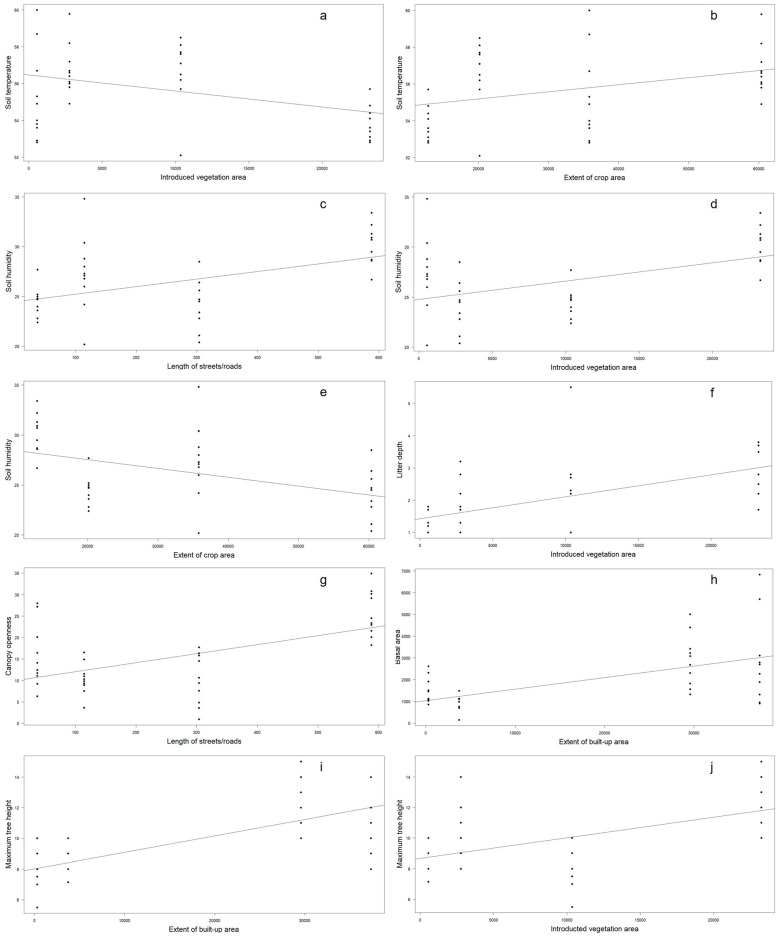
Linear regression analysis between soil temperature and introduced vegetation area and extent of crop area (**a**,**b**); soil humidity and length of streets/roads and introduced vegetation area (**c**,**d**); soil humidity and extent of crop area and introduced vegetation area (**e**,**f**); canopy openness and length of streets/roads and extent of built-up area (**g**,**h**); and maximum tree height and extent of built-up area and introduced vegetation area (**i**,**j**) along the Puebla-Tlaxcala Valley of Mexico. The fitted lines indicate the trend, and only significant relationships are shown (*p* < 0.05).

Soil temperature was negatively related to the extent of areas with introduced vegetation and positively related to the extent of crop areas (r^2^ = 0.15, F = 6.37, *p* = 0.0153; r^2^ = 0.12, F = 5.16, *p* = 0.0281, respectively; Figure 4a,b). The soil humidity was positively related to both the length of streets/roads and the extent of areas with introduced vegetation (r^2^ = 0.21, F = 10.16, *p* = 0.0031; r^2^ = 0.21, F = 10.38, *p* = 0.0024, respectively; Figure 4c,d), and negatively related to the extent of crop areas (r^2^ = 0.20, F = 10.14, *p* = 0.0033; Figure 4e). The litter depth showed a positive relationship with the extent of areas with introduced vegetation (r^2^ = 0.34, F = 19.8, *p* < 0.0001; Figure 4f). The canopy openness was positively related to the length of streets/roads (r^2^ = 0.29, F = 15.57, *p* = 0.0003; Figure 4g). The basal area and the extent of built-up areas were positively related (r^2^ = 0.32, F = 18.33, *p* = 0.0002; Figure 4h). The maximum tree height was positively related to both the extent of built-up areas and the extent of areas with introduced vegetation (r^2^ = 0.53, F = 43, *p* < 0.0001; r^2^ = 0.26, F = 13, *p* = 0.0008, respectively; Figure 4i,j). The rest of the microclimatic, environmental, and vegetation structure variables were not statistically related to urbanization variables (*p* > 0.05).

**Figure 5:**
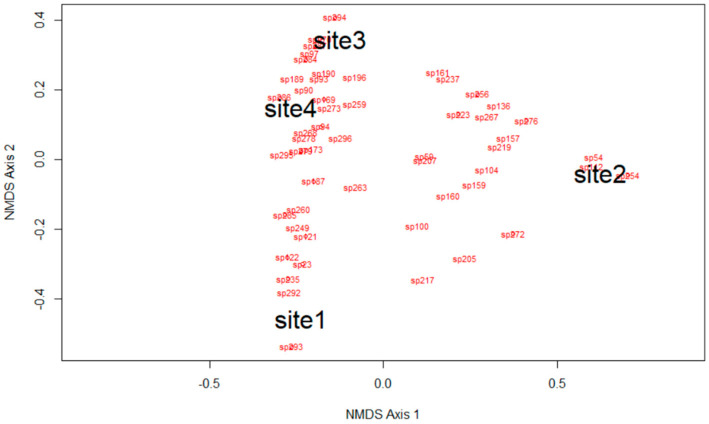
Non-metric Multidimensional Scaling analysis for macrofungal species composition among the four study sites. “sp1, sp2, sp3, sp4…” represents the species recorded in the study area, the numbers of the species correspond to the ID numbers in Table S1.

The Chao-Jaccard similarity index indicated that the percentage of similarity in species composition was higher for Sites 3 and 4 (51%) (Tenancingo-San Nicolás de los Ranchos), which are the less urbanized sites, followed by Sites 1 and 4 (45%) (Flor del Bosque-San Nicolás de los Ranchos), Sites 2 and 4 (43%) (El Aguacate-San Nicolás de los Ranchos), Sites 1 and 3 (42%) (Flor del Bosque-Tenancingo), Sites 2 and 3 (34%) (El Aguacate-Tenancingo), and the least similar were Sites 1 and 2 (19%) (Flor del Bosque-El Aguacate), which are the most urbanized. Correspondingly, the NMDS showed that Sites 3 and 4 (Tenancingo-San Nicolás de los Ranchos) are the closest in terms of similarity in species composition, whereas Sites 1 and 2 (Flor del Bosque-El Aguacate) are the most distant regarding species composition (Figure 5).

**Figure 6:**
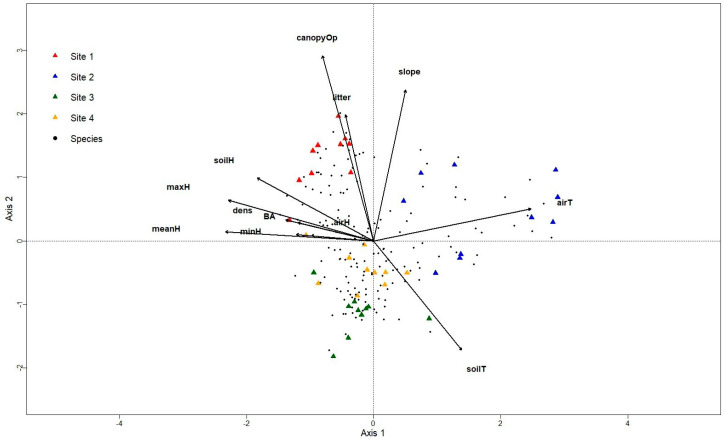
CCA for the macromycete species recorded in the four study sites. Vectors are microclimatic, environmental, and vegetation structure variables: air humidity (airH), soil humidity (soilH), air temperature (airT), soil temperature (soilT), slope, litter depth (litter), canopy openness (canopyOp), mean tree height (meanH), minimum tree height (minH), maximum tree height (maxH), tree density (dens), and basal area (BA).

The CCA for microclimatic, environmental, and vegetation structure variables included air and soil humidity, air and soil temperature, slope, litter depth, canopy openness, tree density, basal area, and minimum, mean, and maximum tree height, and it was performed for the 296 macromycete species recorded in the study area. Axis 1 (eigenvalue = 0.69) and Axis 2 (eigenvalue = 0.60) accounted for 12% and 11% of the explained variance in the relationship between macrofungal species and explanatory variables, respectively. CCA indicated that Site 2 clearly separates from Sites 1, 3, and 4 along axis 1, with air temperature in Site 2 as the main related variable, and humidity and vegetation structure as the main variables explaining the species distribution in Sites 1, 3, and 4. The analysis showed a distinct separation of Site 1 from Sites 3 and 4 along axis 2, mainly due to the relationship of the species in Site 1 with litter depth and canopy openness, and soil temperature in Sites 3 and 4 (Figure 6).

**Figure 7:**
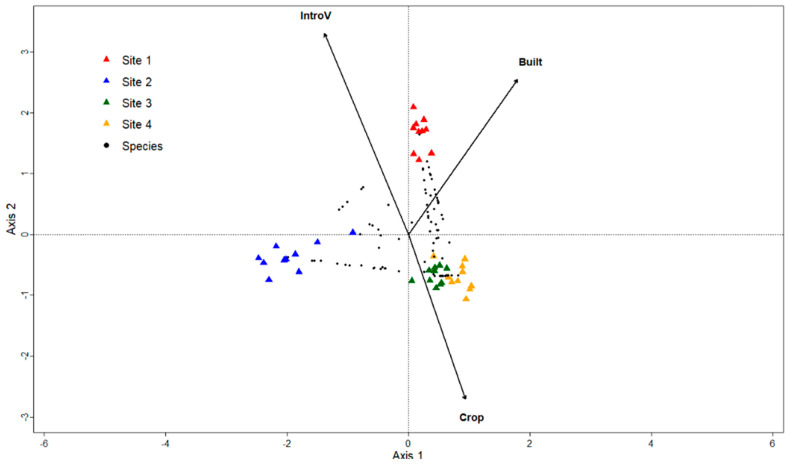
CCA for the macromycete species recorded in the four study sites. Vectors are urbanization variables: extent of built-up area (Built), extent of crop area (Crop), and extent of introduced vegetation area (IntroV).

The CCA for urbanization variables included the extent of crop and built-up areas, extent of introduced vegetation areas, and length of streets and roads, and it was performed for the 296 macromycete species; however, the model retained only three variables. Axis 1 (eigenvalue = 0.62) and Axis 2 (eigenvalue = 0.59) accounted for 36% and 34% of the explained relationship between species distribution and explanatory variables, respectively. There is a clear separation of Sites 1, 3, and 4 from Site 2 along axis 1, with crop areas and built-up areas being the main related variables. Site 1 was clearly separated from Sites 2, 3, and 4 along axis 2, with the species distribution in Site 1 being explained mainly by built-up areas and areas of introduced vegetation, whereas crop areas were the variable more closely associated with Sites 3 and 4 (Figure 7).

## 4. Discussion

**Figure 8:**
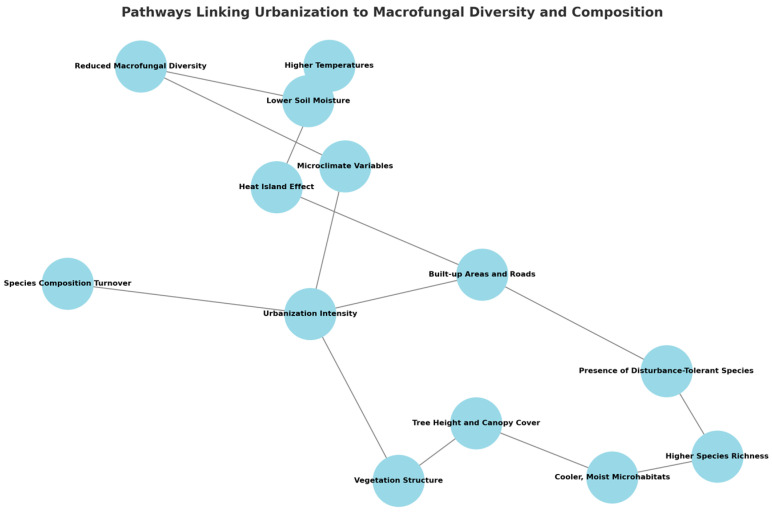
Conceptual model showing how macrofungal diversity and species composition are influenced by the effect of urbanization on microclimate and vegetation structure in the study area. The model illustrates hypothesized causal pathways; node (circle) proximity and link length do not represent effect strength or statistical magnitude.

Mexico ranks third worldwide in ecosystem diversity [59]; however, it is considered a highly urbanized country, with most of the national population living in expanding urban areas [60]. Despite the importance of macromycetes in terrestrial ecosystems, research on these organisms within urbanized landscapes remains limited [51,61], with more represented studies being conducted with birds [62,63], insects [64], and plants [65,66]. The findings of this study suggest that the observed decline in diversity and conspicuous variation in species composition along the urbanization gradient, as well as the high number of species recorded at one of the most urbanized sites, are closely linked to vegetation structure and microclimatic transformations driven by urban development (Figure 8).

Microclimatic factors were found to be strong predictors of species richness. Negative correlations with air and soil temperature, along with positive correlations with soil humidity and tree height, indicate that cooler and wetter habitats favor the proliferation of sporomes, thus underscoring the sensitivity of macromycetes to habitat disturbance and microclimatic changes associated with urban heat islands and the expansion of impervious surfaces [22,25,28,29,30]. The buffering role of tall trees is relevant, as they help regulate temperature, retain soil humidity, and provide abundant woody substrates for xylophagous fungi [21,36]. In contrast, variables such as slope, canopy openness, and litter depth did not show a significant relationship, suggesting that in fragmented and urbanized landscapes, vegetation structure and microclimate are more influential than topographic and environmental factors [24,27].

Interestingly, the results revealed a positive relationship between macromycete species richness and the extent of built-up areas and length of streets/roads along the study area, contrasting with the expected negative relationship. Several studies have documented cases in which urbanized areas host higher species diversity than nearby non-urbanized or rural landscapes, which can be partly explained by cities’ capacity to create heterogeneous environments that support both native and non-native species [67]. The observed relationship between macromycete richness and urbanization variables is likely to be caused by the effect of the Flor del Bosque data in the analysis, since it was the second-most urbanized site and had the highest species richness [119 species]. Flor del Bosque is a protected natural area located within the city of Puebla, surrounded by buildings and streets. Its high species richness highlights the role of habitat protection, continuous tree cover, native vegetation, and heterogeneous microhabitats in maintaining diversity [3,30,31]. Nevertheless, higher species richness in urban settings does not necessarily equate to higher ecological value [68]. It has been observed that disturbance-tolerant macromycetes that exploit novel substrates can be present in urban environments, contributing to the local diversity while simultaneously reducing regional diversity [11]. Moreover, introduced vegetation can play a significant role in increasing species diversity in urban areas, particularly when it enhances habitat heterogeneity and resource availability. In highly transformed landscapes, non-native plant species, including ornamental, fruit, and exotic forest species, may generate novel ecological niches, modify microclimatic conditions, and expand the supply of resources, thereby facilitating the colonization of diverse taxonomic groups [3,68].

Species distribution analyses also revealed the effects of urbanization. The least urbanized sites (Tenancingo and San Nicolás de los Ranchos) showed the highest similarity in species composition, whereas the most urbanized sites (Flor del Bosque and El Aguacate) exhibited the lowest similarity. This pattern reflects an increasing species turnover along the urbanization gradient and is consistent with other studies on macromycetes, birds, and plants [27,52,69], where environmental filters have been observed to favor generalist or disturbance-tolerant species, including non-native ones [5,10]. The results suggest that the effects of soil humidity and tree height on macromycete species distribution are associated with less disturbed sites, while the effects of temperature, canopy openness, built-up areas, and introduced vegetation are linked to species distributed in more urbanized sites. Studies in forests of temperate affinity indicate that the distribution of macromycete species at the local scale can be driven by the variation in vegetation structure as well as other factors affecting temperature, humidity, and the quality and quantity of available resources [21,27,30].

The findings of this study highlight the necessity of urban biodiversity management strategies that prioritize microclimatic buffering, habitat continuity, and the conservation of native tree cover. These measures can help mitigate the negative effects of urban heat and drought while ensuring that macrofungal communities continue to perform essential ecological functions, such as nutrient cycling, population control of animals, plants, and fungi, and nutrient exchange with vascular plants [13,14]. Although field sampling was conducted two years ago, the findings remain highly relevant, as they provide a recent ecological baseline for understanding how macrofungal communities respond to urbanization in the Puebla–Tlaxcala Valley, a rapidly urbanizing region where information on macromycetes remains scarce. The dataset can be a valuable reference point for future monitoring, especially because macrofungal sporocarp production is known to be greatly variable among years and strongly influenced by suitable climatic conditions [70]. For future studies, it would be valuable to include a broader range of vegetation types and examine how urbanization impacts macromycete functional groups. To gain a better understanding of how urbanization affects the structure and ecological functions of macrofungal communities, research should be conducted across different urban ecosystems, incorporating both phylogenetic and functional trait data to assess macromycete diversity and distribution.

## 5. Conclusions

This study suggests that macrofungal communities in the Puebla–Tlaxcala Valley may respond clearly to urbanization, with apparent/detectable shifts in diversity and species composition along the urbanization gradient. The interplay between microclimatic conditions, vegetation structure, and land-use intensity may help explain these patterns, suggesting the sensitivity of macromycetes to environmental alterations associated with urban development.

The findings highlight the ecological value of protected and semi-natural habitats embedded within urban matrices, as these areas can contribute to maintaining substantial macrofungal diversity despite surrounding disturbance. At the same time, urban environments may influence community assembly processes, with potential implications for ecosystem functioning.

Future studies should consider defining functional groups in urban gradients to assess how variations in biotic and abiotic factors affect them, and specific management and conservation strategies should be developed based on the distinct requirements of macrofungal groups and on existing strategies for plants and animals.

## Figures and Tables

**Table 1:** Spearman’s rho correlation coefficient. Correlation between macromycete species richness and explanatory variables in the study area.

Variable	rho	*p*-Value
Soil temperature *	−0.3419	0.0307
Air temperature ***	−0.5277	0.0004
Air humidity	0.2501	0.1195
Soil humidity ***	0.5180	0.0006
Litter depth	−0.0184	0.9098
Canopy openness	−0.0607	0.7097
Slope	−0.0809	0.6196
Basal area	0.1625	0.3162
Minimum height of trees	0.3080	0.0531
Maximum height of trees *	0.3152	0.0475
Mean height of trees **	0.4141	0.0078
Tree density	0.2494	0.1205

* *p* < 0.05, ** *p* < 0.01, *** *p* < 0.001.

## Data Availability

The raw data supporting the conclusions of this article will be made available by the authors on request.

## Supplementary Materials

The following supporting information can be downloaded at: https://www.mdpi.com/article/10.3390/jof12060397/s1, Table S1. Macrofungal species/morphospecies and abundances recorded in the four study sites along the Puebla-Tlaxcala Valley of Mexico.

## References

[1] Antrop M. (2004). Landscape change and the urbanization process in Europe. Landsc. Urban Plan..

[2] Hansen A.J., Knight R.L., Marzluff J.M., Powell S., Brown K., Gude P.H., Jones K. (2005). Effects of exurban development on biodiversity: Patterns, mechanisms, and research needs. Ecol. Appl..

[3] Kowarik I. (2011). Novel urban ecosystems, biodiversity, and conservation. Environ. Pollut..

[4] Alberti M. (2015). Eco-evolutionary dynamics in an urbanizing planet. Trends Ecol. Evol..

[5] Cousins J.R., Hope D., Gries C., Stutz J.C. (2003). Preliminary assessment of arbuscular mycorrhizal fungal diversity and community structure in an urban ecosystem. Mycorrhiza.

[6] McPhearson T., Pickett S.T.A., Grimm N.B., Niemelä J., Alberti M., Elmqvist T., Qureshi S. (2016). Advancing urban ecology toward a science of cities. BioScience.

[7] United Nations, Department of Economic and Social Affairs, Population Division (2018). The World’s Cities in 2018: Data Booklet (ST/ESA/SER.A/417).

[8] UN-Habitat (2022). World Cities Report 2022: Envisaging the Future of Cities.

[9] (2025). Worldometers. World Population. https://www.worldometers.info/.

[10] MacGregor-Fors I., Avendaño-Reyes S., Bandala V.M., Chacón-Zapata S., Díaz-Toribio M., González-García F., Escobar F. (2015). Multitaxonomic diversity patterns in a neotropical green city: A rapid biological assessment. Urban Ecosyst..

[11] Avis P.G., Mueller G.M., Lussenhop J. (2016). Monitoring fungi in ecological restorations of coastal Indiana, U.S.A. Restor. Ecol..

[12] Caiafa M.V., Gómez-Hernández M., Williams-Linera G., Ramírez-Cruz V. (2017). Functional diversity of macromycete communities along an environmental gradient in a Mexican seasonally dry tropical forest. Fungal Ecol..

[13] Bahram M., Netherway T. (2022). Fungi as mediators linking organisms and ecosystems. FEMS Microbiol. Rev..

[14] Niego A.G.T. (2023). The contribution of fungi to the global economy. Fungal Divers..

[15] Lonsdale D., Pautasso M., Holdenrieder O. (2008). Wood-decaying fungi in the forest: Conservation needs and management options. Eur. J. For. Res..

[16] Tedersoo L., May T.W., Smith M.E. (2010). Ectomycorrhizal lifestyle in fungi: Global diversity, distribution, and evolution of phylogenetic lineages. Mycorrhiza.

[17] Bever J.D., Platt T.G., Morton E.R. (2012). Microbial population and community dynamics on plant roots and their feedbacks on plant communities. Annu. Rev. Microbiol..

[18] Baldrian P., Bell-Dereske L., Lepinay C., Větrovský T., Kohout P. (2022). Fungal communities in soils under global change. Stud. Mycol..

[19] Mueller G.M., Schmit J.P., Leacock P.R., Buyck B., Cifuentes J., Desjardin D.E., Wu Q. (2007). Global diversity and distribution of macrofungi. Biodivers. Conserv..

[20] Aguirre-Acosta C.E., Ulloa M., Aguilar S., Cifuentes J., Valenzuela R. (2014). Biodiversidad de hongos en México. Rev. Mex. Biodivers..

[21] Pérez-Rosas M., Gómez-Hernández M., Gándara E. (2022). Variation in macrofungal diversity and species composition across different vegetation types in Oaxaca, Mexico. Bot. Sci..

[22] Brown N., Bhagwat S., Watkinson S. (2006). Macrofungal diversity in fragmented and disturbed forests of the Western Ghats of India. J. Appl. Ecol..

[23] Gómez-Hernández M., Williams-Linera G. (2011). Diversity of macromycetes in tropical cloud forests in Veracruz, Mexico. Botany.

[24] Gómez-Hernández M., Williams-Linera G., Guevara R., Lodge J. (2012). Patterns of macromycete community assemblage along an elevation gradient: Options for fungal gradient and metacommunity analyses. Biodivers. Conserv..

[25] Zhang Y., Zhou D.Q., Zhao Q., Zhou T.X., Hyde K.D. (2010). Diversity and ecological distribution of macrofungi in the Laojun Mountain region, southwestern China. Biodivers. Conserv..

[26] Newbound M., McCarthy M.A., Lebel T. (2010). Fungi and the urban environment: A review. Landsc. Urban Plan..

[27] Gómez-Hernández M., Ramírez-Antonio K.G., Gándara E. (2019). Ectomycorrhizal and wood-decay macromycete communities along development stages of managed Pinus patula stands in Southwest Mexico. Fungal Ecol..

[28] Arnfield A.J. (2003). Two decades of urban climate research: A review of turbulence, exchanges of energy and water, and the urban heat island. Int. J. Climatol..

[29] Boone C.G., Fragkias M. (2012). Urbanization and Sustainability: Linking Urban Ecology, Environmental Justice and Global Environmental Change.

[30] Ferris R., Peace A.J., Newton A.C. (2000). Macrofungal communities of lowland Scots pine and Norway spruce plantations in England: Relationships with site factors and stand structure. For. Ecol. Manag..

[31] Rubino D.L., McCarthy B.C. (2003). Evaluation of coarse woody debris and forest vegetation across topographic gradients in a southern Ohio forest. For. Ecol. Manag..

[32] Instituto Nacional de Estadística y Geografía (INEGI) (2020). Censo de Población y Vivienda 2020. https://www.inegi.org.mx/programas/ccpv/2020/.

[33] Lemin M., Vázquez A., Chacón S. (2010). Etnomicología y comercialización de hongos en mercados de tres poblados del noreste del estado de Puebla, México. Brenesia.

[34] Pérez-López R.I., Mata G., Aragón García A., Jiménez García D., Romero-Arenas O. (2015). Diversidad de hongos silvestres comestibles del cerro El Pinal, municipio de Acajete, Puebla, México. Ecosistemas Recur. Agropecu..

[35] Contreras-Cortés L.E.U., Vázquez-García A., Ruan-Soto F. (2018). Etnomicología y venta de hongos en un mercado del noroeste del estado de Puebla, México. Sci. Fungorum.

[36] Vázquez S., Valenzuela R., Castillo R.F.d. (2016). Macromicetos lignícolas de la Sierra Norte de Puebla, México, con notas sobre su distribución altitudinal. Acta Botánica Mex..

[37] Sánchez Flores M., Martínez-Pineda M., Raymundo T. (2021). Ionomidotis mesophilus (Ascomycota, Cordieritidaceae), una especie nueva del bosque de niebla en México. Acta Botánica Mex..

[38] Reyes-López R.C., Montoya A., Kong A., Caballero-Nieto J. (2020). Folk classification of wild mushrooms from San Isidro Buensuceso, Tlaxcala, Central Mexico. J. Ethnobiol. Ethnomed..

[39] Ramírez-Terrazo A., Montoya A., Garibay-Orijel R., Caballero-Nieto J., Kong-Luz A., Méndez-Espinoza C. (2021). Breaking the paradigms of residual categories and neglectable importance of non-used resources: The vital traditional knowledge of non-edible mushrooms. J. Ethnobiol. Ethnomed..

[40] Williams-Linera G., Domínguez-Gastelú V., García-Zurita M.E. (1998). Microenvironment and floristics of different edges in a fragmented tropical rainforest. Conserv. Biol..

[41] Breitenbach J., Kränzlin F. (1991). Fungi of Switzerland. Vol. 3: Boletes and Agarics. Part 1: Strobilomycetaceae, Boletaceae, Paxillaceae, Gomphidiaceae, Hygrophoraceae, Tricholomataceae, Polyporaceae (lamellate).

[42] Breitenbach J., Kränzlin F. (1995). Fungi of Switzerland. Vol. 4: Agarics. Part 2: Entolomataceae, Pluteaceae, Amanitaceae, Agaricaceae, Coprinaceae, Strophariaceae.

[43] Breitenbach J., Kränzlin F. (2000). Fungi of Switzerland. Vol. 5: Agarics. Part 3: Cortinariaceae.

[44] Guzmán G. (1977). Identificación de los Hongos: Comestibles, Venenosos, Alucinantes y Destructores de la Madera.

[45] Læssøe T., Petersen J. (2019). Fungi of Temperate Europe 1–2.

[46] Largent D.L. (1986). How to Identify Mushrooms to Genus I: Macroscopic Features.

[47] Largent D.L., Thiers H.D. (1977). How to Identify Mushrooms to Genus II: Field Identification of Genera.

[48] Pegler D.N. (1977). A Preliminary Agaric Flora of East Africa.

[49] Pegler D.N. (1983). Agaric flora of the Lesser Antilles.

[50] Pegler D.N. (1986). Agaric flora of Sri Lanka.

[51] Gómez-Hernández M., Avendaño-Villegas E., Toledo-Garibaldi M., Gándara E. (2021). Impact of urbanization on functional diversity in macromycete communities along an urban ecosystem in Southwest Mexico. PeerJ.

[52] McDonnell M.J., Hahs A.K. (2008). The use of gradient analysis studies in advancing understanding of the ecology of urbanizing landscapes. Landsc. Ecol..

[53] Esri (2015). ArcGIS Desktop.

[54] Jost L. (2006). Entropy and diversity. Oikos.

[55] Jost L. (2007). Partitioning diversity into independent alpha and beta components. Ecology.

[56] Chao A., Chazdon R.L., Colwell R.K., Shen T.-J. (2005). A new statistical approach for assessing similarity of species composition with incidence and abundance data. Ecol. Lett..

[57] R Core Team (2017). R: A Language and Environment for Statistical Computing. https://www.R-project.org/.

[58] Python Software Foundation (2023). Python.

[59] CONABIO (2008). Capital Natural de México. Volumen I: Conocimiento Actual de la Biodiversidad.

[60] United Nations Human Settlements Programme (UN-Habitat) (2025). Urbanization in Mexico: Building Inclusive & Sustainable Cities. https://unhabitat.org/mexico.

[61] Pérez Silva E. (2018). Hongos de zonas urbanas: Ciudad de México y Estado de México. Sci. Fungorum.

[62] Bell M.B. (2008). Receiver identity modifies begging intensity independent of need in banded mongoose (*Mungos mungo*) pups. Behav. Ecol..

[63] Marzluff J.M. (2016). A decadal review of urban ornithology and a prospectus for the future. Ibis.

[64] Lagucki E., Burdine J.D., McCluney K.E. (2017). Urbanization alters communities of flying arthropods in parks and gardens of a medium-sized city. PeerJ.

[65] Falfán I., MacGregor-Fors I. (2016). Woody neotropical streetscapes: A case study of tree and shrub species richness and composition in Xalapa. Madera Bosques.

[66] Jara-Toto E., Armenta-Montero S., Aquino-Zapata A.M., Carvajal Hernández C.I. (2023). Diversidad y estructura de la vegetación leñosa en cuatro bosques urbanos de la zona conurbada Xalapa-Banderilla, Veracruz, México. Acta Bot. Mex..

[67] Shochat E., Warren P.S., Faeth S.H., McIntyre N.E., Hope D. (2006). From patterns to emerging processes in mechanistic urban ecology. Trends Ecol. Evol..

[68] Aronson M.F.J., La Sorte F.A., Nilon C.H., Katti M., Goddard M.A., Lepczyk C.A., Warren P.S., Williams N.S.G., Cilliers S., Clarkson B. (2014). A global analysis of the impacts of urbanization on bird and plant diversity reveals key anthropogenic drivers. Proc. R. Soc. B Biol. Sci..

[69] Ortega-Álvarez R., Rodríguez-Correa H.A., MacGregor-Fors I. (2011). Trees and the city: Diversity and composition along a neotropical gradient of urbanization. Int. J. Ecol..

[70] Ponce Á., Alday J.G., Martínez de Aragón J., Collado E., Morera A., Bonet J.A., de-Miguel S. (2022). Environmental drivers shaping the macrofungal sporocarp community in Mediterranean Quercus ilex stands. For. Ecol. Manag..

