# Supplementary material for: Macromycetes Under Pressure: Diversity and Species Composition Across an Urbanization Gradient in the Puebla-Tlaxcala Valley of Mexico
Source: J Fungi (Basel). 2026 May 30;12(6):397. doi: 10.3390/jof12060397 (PMC13302147; doi:10.3390/jof12060397)
Supplement: Supplementary file 1 [file jof-12-00397-s001.zip › jof-4320968-supplementary.pdf]

Table S1. Macrofungal species/morphospecies and abundances recorded in the four study sites along the Puebla-Tlaxcala Valley of Mexico.

ID number	Species	Site 1	Site 2	Site 3	Site 4
sp1	*Agaricus albolutescens* Zeller	1	0	0	0
sp2	*Agaricus arvensis* Schaeff.	0	0	0	1
sp3	*Agaricus augustus* Fr.	0	1	0	0
sp4	*Agaricus benesii* (Pilát) Pilát	0	2	0	0
sp5	*Agaricus benzodorus* Heinem. & Gooss.-Font.	0	1	0	0
sp6	*Agaricus bernardii* Quél.	3	0	1	0
sp7	*Agaricus brunneolus* (J.E. Lange) Pilát	0	0	0	1
sp8	*Agaricus campestris* L.	4	0	0	0
sp9	*Agaricus cervinoculus* Palestina-Villa, Medel, Gar.-Orijel, Linda J. Chen & L.A. Parra	1	0	0	0
sp10	*Agaricus* sp.1	0	1	0	0
sp11	*Agaricus* sp.2	0	1	0	0
sp12	*Agaricus* sp.3	0	0	0	1
sp13	*Agaricus* sp.4	0	0	1	0
sp14	Agaricaceae 1	0	0	0	1
sp15	Agaricaceae 2	1	0	0	0
sp16	Agaricaceae 3	0	0	1	0
sp17	Agaricaceae 4	0	0	0	2
sp18	Agaricaceae 5	1	0	0	0
sp19	Agaricaceae 6	1	0	0	0

sp20	Agaricaceae 7	1	0	0	0
sp21	Agaricaceae 8	1	0	0	0
sp22	Agaricaceae 9	0	0	3	1
sp23	Agaricaceae 10	0	1	0	0
sp24	Agaricaceae 11	2	0	4	0
sp25	Agaricaceae 12	1	0	0	1
sp26	Agaricaceae 13	0	0	1	0
sp27	Agaricaceae 14	0	0	1	0
sp28	Agaricaceae 15	0	0	1	0
sp29	Agaricaceae 16	0	0	1	0
sp30	Agaricaceae 17	0	0	0	1
sp31	Agaricaceae 18	3	0	1	0
sp32	Agaricaceae 19	1	0	0	0
sp33	Agaricaceae 20	0	0	0	1
sp34	Agaricaceae 21	0	0	2	0
sp35	Agaricaceae 22	2	0	1	0
sp36	Agaricaceae 23	0	0	1	0
sp37	Agaricaceae 24	2	0	0	0
sp38	Agaricaceae 25	1	0	0	0
sp39	Agaricaceae 26	0	0	1	0
sp40	Agaricaceae 27	0	1	0	0
sp41	Agaricaceae 28	0	1	0	0
sp42	Agaricaceae 29	0	0	0	1
sp43	Agaricaceae 30	0	1	0	0
sp44	Agaricaceae 31	0	0	1	0
sp45	Agaricaceae 32	1	0	0	0
sp46	Agaricaceae 33	1	0	0	0
sp47	Agaricaceae 34	0	0	1	0

sp48	Agaricaceae 35	1	0	0	0
sp49	Agaricaceae 36	0	1	0	0
sp50	Agaricaceae 37	1	0	0	0
sp51	Agaricaceae 38	0	0	1	0
sp52	Agaricaceae 39	1	0	0	0
sp53	Agaricaceae 40	0	0	1	0
sp54	Agaricaceae 41	0	0	1	0
sp55	Agaricaceae 42	0	1	0	0
sp56	Agaricaceae 43	0	0	1	0
sp57	Agaricaceae 44	1	0	0	0
sp58	Agaricaceae 45	0	4	1	0
sp59	Agaricaceae 46	0	0	2	0
sp60	Agaricaceae 47	0	1	0	0
sp61	Agaricaceae 48	0	0	0	1
sp62	Agaricaceae 49	1	2	0	4
sp63	Agaricaceae 50	0	0	1	0
sp64	Agaricaceae 51	1	0	0	0
sp65	Agaricaceae 52	0	0	1	0
sp66	Agaricaceae 53	0	0	0	1
sp67	Agaricaceae 54	0	0	1	0
sp68	Agaricaceae 55	2	0	0	1
sp69	Agaricaceae 56	1	0	0	0
sp70	Agaricaceae 57	2	0	0	0
sp71	Agaricaceae 58	0	0	0	1
sp72	Agaricaceae 59	0	0	1	0
sp73	Agaricaceae 60	1	0	0	0
sp74	Agaricaceae 61	1	0	0	0
sp75	Agaricaceae 62	1	0	0	0

sp76	Agaricaceae 63	0	0	1	0
sp77	Agaricaceae 64	0	2	0	0
sp78	Agaricaceae 65	0	0	1	0
sp79	Agaricaceae 66	1	0	0	0
sp80	Agaricaceae 67	1	0	0	0
sp81	Agaricaceae 68	0	0	0	2
sp82	Agaricaceae 69	0	0	0	1
sp83	*Agrocybe* sp.1	0	0	0	3
sp84	*Amanita bisporigera* G.F. Atk.	0	0	8	0
sp85	*Amanita flavoconia* G.F. Atk.	0	0	4	3
sp86	*Amanita gemmata* (Fr.) Bertill.	0	0	0	1
sp87	*Amanita laurae* Guzmán & Ram.-Guill.	1	0	0	0
sp88	*Amanita novinupta* Tulloss & J.E. Lindgr.	2	1	0	1
sp89	*Amanita rubescens* Pers.	2	0	0	1
sp90	*Amanita tuza* Guzmán	0	0	0	1
sp91	*Amanita* sp.1	0	0	1	0
sp92	*Amanita* sp.2	0	0	0	1
sp93	*Amanita* sp.3	1	0	4	4
sp94	*Amanita* sp.4	1	0	0	0
sp95	*Amanita* sp.5	3	0	14	3
sp96	*Amanita* sp.6	1	0	2	0
sp97	*Amanita* sp.7	0	0	2	0
sp98	*Amanita* sp.8	0	0	2	0
sp99	*Amanita* sp.9	0	0	3	0

sp100	*Astraeus hygrometricus* (Pers.) Morgan	4	8	3	4
sp101	*Astraeus* sp.1	0	0	1	0
sp102	*Boletus aereus* Bull.	0	1	0	0
sp103	*Boletus* aff. *edulis*	0	5	0	0
sp104	*Boletus reticulatus* Schaeff.	1	0	0	0
sp105	*Boletus speciosus* Frost	0	4	0	0
sp106	*Boletus* sp.1	0	0	1	1
sp107	*Boletus* sp.2	0	3	0	0
sp108	*Boletus* sp.3	0	1	0	0
sp109	*Boletus* sp.4	0	5	0	1
sp110	*Boletus* sp.5	0	2	0	0
sp111	*Boletus* sp.6	0	1	0	0
sp112	*Calvatia* sp.1	0	0	3	0
sp113	*Cantharellus* aff. *cibarius*	0	0	1	0
sp114	*Coltricia perennis* (L.) Murrill	0	0	0	1
sp115	*Coltricia* sp.1	0	0	0	7
sp116	*Coprinellus disseminatus* (Pers.) J.E. Lange	4	0	0	2
sp117	*Coprinopsis atramentaria* (Bull.) Redhead, Vilgalys & Moncalvo	0	0	2	0
sp118	*Coprinus calyptratus* Peck	0	0	0	1
sp119	*Coprinus comatus* (O.F. Müll.) Pers.	1	0	0	0

sp120	*Coprinus mexicanus* Murrill	1	0	3	0
sp121	*Coprinus silvaticus* Peck	0	0	0	1
sp122	*Coprinus* sp.1	2	0	1	0
sp123	*Coprinus* sp.2	1	0	0	1
sp124	*Coprinus* sp.3	1	0	0	0
sp125	*Coprinus* sp.4	1	0	0	0
sp126	*Coprinus* sp.5	0	0	0	1
sp127	*Coprinus* sp.6	0	0	1	0
sp128	*Coprinus* sp.7	0	1	0	0
sp129	*Cortinarius alboviolaceus* (Pers.) Zaw.	1	0	0	0
sp130	*Cortinarius caerulescens* (Schaeff.) Fr.	0	0	3	0
sp131	*Cortinarius glaucopus* (Schaeff.) Gray	0	0	2	0
sp132	*Cortinarius mexicanus* Murrill	0	2	3	0
sp133	*Cortinarius* sp.1	1	0	0	1
sp134	*Cortinarius* sp.2	0	1	0	0
sp135	*Cortinarius* sp.3	0	0	1	0
sp136	*Cortinarius* sp.4	0	0	0	1
sp137	*Cortinarius* sp.5	0	0	1	0
sp138	*Craterellus tubaeformis* (Fr.) Quél.	0	0	0	1
sp139	*Crepidotus mollis* (Schaeff.) Staude	0	0	1	0
sp140	*Crepidotus* sp.1	1	0	0	0

sp141	*Crepidotus* sp.2	0	0	0	4
sp142	*Crepidotus* sp.3	1	0	0	0
sp143	*Crepidotus* sp.4	3	0	0	1
sp144	*Crepidotus* sp.5	2	0	0	0
sp145	*Crepidotus* sp.6	1	0	0	0
sp146	*Dacrymyces* sp.1	0	0	1	0
sp147	*Daldinia* sp.1	3	0	0	1
sp148	*Deconica coprophila* (Bull.) P. Karst.	0	0	0	2
sp149	*Entoloma* sp.1	1	0	1	0
sp150	*Entoloma* sp.2	1	0	0	0
sp151	*Entoloma* sp.3	0	1	0	0
sp152	*Fuscoporia gilva* (Schwein.) T. Wagner & M. Fisch.	2	0	0	2
sp153	*Ganoderma* sp.1	0	0	0	2
sp154	*Geastrum saccatum* Fr.	0	3	1	2
sp155	*Gymnopus* sp.1	0	0	0	1
sp156	*Gymnopus* sp.2	5	6	3	2
sp157	*Gymnopus* sp.3	1	1	0	1
sp158	*Gymnopus* sp.4	0	2	7	0
sp159	*Gymnopus* sp.5	0	0	0	1
sp160	*Hebeloma* sp.1	1	0	1	0
sp161	*Helvella crispa* Bull.	0	0	2	0
sp162	*Helvella lacunosa* Afzel.	0	0	2	2
sp163	*Hydnellum* sp.1	0	0	1	0
sp164	*Hydnum repandum* L.	2	0	0	0
sp165	*Hygrocybe* sp.1	1	0	0	0

sp166	*Hygrocybe conica* (Schaeff.) P. Kumm.	1	0	3	0
sp167	*Hygrophorus* sp.1	0	0	3	0
sp168	*Hygrophorus russula* (Schaeff. ex Fr.) Bataille	0	0	3	0
sp169	*Hymenochaete cinnamomea* (Pers.) Bres.	0	0	0	1
sp170	*Hymenochaete curtisii* (Berk.) Morgan	0	1	0	0
sp171	*Hymenochaete episphaeria* (Schwein. ex Fr.) Massee	0	0	0	4
sp172	*Hymenochaete fulva* Burt	0	1	2	0
sp173	*Hymenochaete luteobadia* (Fr.) Höhn. & Litsch.	0	1	0	0
sp174	*Hymenochaete* sp.1	0	0	0	1
sp175	*Hymenochaete* sp.2	3	0	4	1
sp176	*Hymenochaete* sp.3	0	0	2	1
sp177	*Hymenochaete* sp.4	0	0	0	1
sp178	*Hymenochaete* sp.5	0	0	1	0
sp179	*Hymenochaete* sp.6	0	0	1	0
sp180	*Hymenochaete* sp.7	0	0	1	0
sp181	*Hypholoma* sp.1	0	0	0	1
sp182	*Hypomyces* sp.1	1	0	0	0
sp183	*Hypomyces lactifluorum* (Schwein.) Tul. & C. Tul.	1	0	0	0
sp184	*Inocybe* sp.1	1	0	1	0
sp185	*Inocybe* sp.2	0	0	1	0

sp186	*Laccaria amethystina* Cooke	0	0	1	3
sp187	*Laccaria bicolor* (Maire) P.D. Orton	0	1	10	14
sp188	*Laccaria laccata* (Scop.) Cooke	0	0	1	0
sp189	*Laccaria* sp.1	0	0	0	1
sp190	*Laccaria* sp.2	0	1	0	0
sp191	*Lactarius chrysorrheus* Fr.	0	1	5	5
sp192	*Lactarius indigo* (Schwein.) Fr.	0	1	0	0
sp193	*Lactarius salmonicolor* R. Heim & Leclair	0	1	0	0
sp194	*Lactarius* sp.1	1	0	0	0
sp195	*Lentinus crinitus* (L.) Fr.	0	1	1	1
sp196	*Lentinus* sp.1	1	0	0	0
sp197	*Lepiota clypeolaria* (Bull.) P. Kumm.	5	0	0	0
sp198	*Lepiota* sp.1	0	0	3	3
sp199	*Lepiota* sp.2	1	1	0	0
sp200	*Lepista nuda* (Bull.) Cooke	9	5	0	0
sp201	*Lepista* sp.1	0	0	0	1
sp202	*Lepista* sp.2	0	1	1	0
sp203	*Leucoagaricus* sp.1	0	0	2	0
sp204	*Lycoperdon perlatum* Pers.	0	0	1	1
sp205	*Lycoperdon* sp.1	1	1	1	1
sp206	*Lycoperdon* sp.2	1	0	1	1
sp207	*Lycoperdon* sp.3	1	0	0	0

sp208	*Lyophyllum* sp.1	0	0	4	0
sp209	*Marasmius* sp.1	0	0	1	1
sp210	*Marasmius* sp.2	0	1	0	0
sp211	*Marasmius* sp.3	6	0	0	0
sp212	*Marasmius* sp.4	1	0	0	0
sp213	*Marasmius* sp.5	0	0	0	1
sp214	*Marasmius* sp.6	3	1	0	0
sp215	*Marasmius* sp.7	5	0	0	0
sp216	*Marasmius* sp.8	0	1	0	1
sp217	*Marasmius* sp.9	0	0	1	1
sp218	*Marasmius* sp.10	1	0	0	0
sp219	*Marasmius* sp.11	0	6	0	0
sp220	*Marasmius* sp.12	0	1	1	1
sp221	*Mycena* sp.1	1	0	0	1
sp222	*Mycena* sp.2	0	0	0	2
sp223	*Mycena* sp.3	0	0	0	4
sp224	*Mycena* sp.4	0	0	2	2
sp225	*Mycena* sp.5	0	0	0	2
sp226	*Mycena* sp.6	1	0	0	0
sp227	*Mycena* sp.7	0	0	0	1
sp228	*Panus conchatus* (Bull.) Fr.	0	0	0	1
sp229	*Panus* sp.1	0	0	0	1
sp230	*Panus* sp.2	0	0	1	0
sp231	*Panus* sp.3	1	0	0	0
sp232	*Peziza* sp.1	3	0	0	1
sp233	*Peziza* sp.2	0	1	0	0
sp234	*Peziza* sp.3	0	1	3	0

sp235	*Peziza* sp.4	0	0	0	1
sp236	*Phellinus* sp.1	1	0	0	1
sp237	*Phellodon niger* (Fr.) P. Karst.	3	0	0	0
sp238	*Pisolithus* sp.1	0	2	0	0
sp239	*Pluteus* sp.1	1	0	1	1
sp240	*Pluteus* sp.2	0	0	1	0
sp241	*Polyporus* sp.1	0	0	0	1
sp242	*Polyporus* sp.2	0	0	0	1
sp243	*Polyporus* sp.3	0	0	0	1
sp244	*Polyporus* sp.4	0	1	0	0
sp245	*Psathyrella* sp.1	3	0	1	1
sp246	*Ramaria* sp.1	0	0	0	1
sp247	*Ramaria* sp.2	0	2	0	0
sp248	*Ramaria* sp.3	2	0	0	0
sp249	*Ramaria* sp.4	0	2	0	0
sp250	*Rubroboletus* sp.1	0	1	0	0
sp251	*Russula brevipes* Peck	0	2	2	1
sp252	*Russula foetens* Pers.	2	0	3	2
sp253	*Russula pectinata* Fr.	0	0	2	0
sp254	*Russula pectinatoides* Peck	0	0	6	2
sp255	*Russula vesicatoria* Burl.	0	0	1	0
sp256	*Russula xerampelina* (Schaeff.) Fr.	4	4	0	0
sp257	*Russula* sp.1	0	0	1	0
sp258	*Russula* sp.2	0	1	2	0
sp259	*Russula* sp.3	0	0	0	2

sp260	*Russula* sp.4	1	0	0	0
sp261	*Russula* sp.5	0	1	1	6
sp262	*Russula* sp.6	7	0	3	3
sp263	*Russula* sp.7	1	0	0	0
sp264	*Russula* sp.8	0	0	0	1
sp265	*Russula* sp.9	12	3	6	8
sp266	*Russula* sp.10	5	0	0	0
sp267	*Russula* sp.11	1	0	0	0
sp268	*Russula* sp.12	0	0	0	1
sp269	*Scleroderma* sp.1	3	1	8	9
sp270	*Sebacina* sp.1	4	0	0	0
sp271	*Sparassis* sp.1	1	0	0	0
sp272	*Stereum ostrea*	0	1	1	0
sp273	*Stereum* sp.1	0	0	2	0
sp274	*Strobilomyces strobilaceus*	3	0	4	3
sp275	*Strobilomyces* sp.1	1	0	1	1
sp276	*Suillus* sp.1	0	0	1	0
sp277	*Trametes* sp.1	1	0	0	0
sp278	*Trametes* sp.2	0	0	0	1
sp279	*Tremella* sp.1	1	0	0	0
sp280	*Phaeotremella foliacea* (Pers.) Wedin, J.C. Zamora & Millanes	0	0	1	1
sp281	*Tremella fuciformis* Berk.	1	0	0	1
sp282	*Tremella mesentérica* Retz.	0	0	0	1
sp283	*Tricholoma equestre* (L.) P. Kumm.	3	0	0	0

sp284	*Tricholoma* sp.1	0	0	2	1
sp285	*Tricholoma* sp.2	0	0	1	0
sp286	*Tricholoma* sp.3	0	0	1	0
sp287	*Tricholoma* sp.4	1	0	0	0
sp288	*Tricholoma* sp.5	0	0	3	0
sp289	*Tricholoma* sp.6	7	0	0	0
sp290	*Tylopilus* sp.1	4	0	0	0
sp291	*Tylopilus* sp.2	1	0	0	0
sp292	*Xerocomus chrysenteron* (Bull.) Quél.	4	0	0	1
sp293	*Xerocomus* sp.1	1	0	0	0
sp294	*Xerocomus* sp.2	0	0	1	0
sp295	*Xeromphalina tenuipes* (Schwein.) A.H. Sm.	1	0	0	3
sp296	*Xylaria hypoxylon* (L.) Grev.	3	2	1	14
